# Assessing telomeric DNA content in pediatric cancers using whole-genome sequencing data

**DOI:** 10.1186/gb-2012-13-12-r113

**Published:** 2012-12-11

**Authors:** Matthew Parker, Xiang Chen, Armita Bahrami, James Dalton, Michael Rusch, Gang Wu, John Easton, Nai-Kong Cheung, Michael Dyer, Elaine R Mardis, Richard K Wilson, Charles Mullighan, Richard Gilbertson, Suzanne J Baker, Gerard Zambetti, David W Ellison, James R Downing, Jinghui Zhang

**Affiliations:** 1 Department of Computational Biology, St Jude Children's Research Hospital, 262 Danny Thomas Place, Memphis, TN 38105, USA; 2 Department of Pathology, St Jude Children's Research Hospital, 262 Danny Thomas Place, Memphis, TN 38105, USA; 3 The Pediatric Cancer Genome Project, St Jude Children's Research Hospital, 262 Danny Thomas Place, Memphis, TN 38105, USA; 4 Department of Pediatrics, Memorial Sloan-Kettering Cancer Center, 1275 York Avenue, New York, NY 10065, USA; 5 Department of Developmental Neurobiology, St Jude Children's Research Hospital, 262 Danny Thomas Place, Memphis, TN 38105, USA; 6 The Genome Center at Washington University, Washington University, 4444 Forest Park Ave, St Louis, Missouri 63108, USA; 7 Department of Genetics, Washington University, 4444 Forest Park Ave, St Louis, Missouri 63108, USA; 8 Department of Biochemistry, St Jude Children's Research Hospital, 262 Danny Thomas Place, Memphis, TN 38105, USA

## Abstract

**Background:**

Telomeres are the protective arrays of tandem TTAGGG sequence and associated proteins at the termini of chromosomes. Telomeres shorten at each cell division due to the end-replication problem and are maintained above a critical threshold in malignant cancer cells to prevent cellular senescence or apoptosis. With the recent advances in massive parallel sequencing, assessing telomere content in the context of other cancer genomic aberrations becomes an attractive possibility. We present the first comprehensive analysis of telomeric DNA content change in tumors using whole-genome sequencing data from 235 pediatric cancers.

**Results:**

To measure telomeric DNA content, we counted telomeric reads containing TTAGGGx4 or CCCTAAx4 and normalized to the average genomic coverage. Changes in telomeric DNA content in tumor genomes were clustered using a Bayesian Information Criterion to determine loss, no change, or gain. Using this approach, we found that the pattern of telomeric DNA alteration varies dramatically across the landscape of pediatric malignancies: telomere gain was found in 32% of solid tumors, 4% of brain tumors and 0% of hematopoietic malignancies. The results were validated by three independent experimental approaches and reveal significant association of telomere gain with the frequency of somatic sequence mutations and structural variations.

**Conclusions:**

Telomere DNA content measurement using whole-genome sequencing data is a reliable approach that can generate useful insights into the landscape of the cancer genome. Measuring the change in telomeric DNA during malignant progression is likely to be a useful metric when considering telomeres in the context of the whole genome.

## Background

Telomeres are the protective caps at the ends of chromosomes and are composed of telomeric DNA repeats, TTAGGG, and associated proteins. The telomeres are critical for genomic stability, as they prevent chromosome ends from being recognized as double strand breaks; they prevent end-to-end chromosome fusions and help maintain replicative competence. Telomere length varies widely among individuals at birth [1] and decreases with each cell division since the DNA replication machinery is unable to replicate chromosome ends ('end-replication problem'). Telomere attrition inevitably reaches a critical point at which cellular senescence or apoptosis is triggered [2]. Approximately 85% of cancers [3] escape the cellular crisis caused by telomere shortening by activating telomerase, an enzyme that catalyzes the synthesis of telomeric DNA from an RNA template. An alternative mechanism to lengthen telomeres has also been observed in a small number of malignancies termed 'alternative lengthening of telomeres' (ALT) [4]. This mechanism operates in a telomerase-independent fashion and is characterized by the production of long, heterogeneous telomeres [5] that can be identified as large bright nuclear foci by fluorescence *in situ *hybridization (FISH) [6].

A number of experimental methods have been used to measure telomere length. Telomere restriction fragment (TRF) analysis involves digesting a large quantity of genomic DNA (1.5 to 2 µg) with enzymes that cut near the ends of the chromosomes. Southern blotting of this DNA with a telomere probe detects the sizes of the restriction fragments generated and thereby provides an average telomere length estimation. FISH can be useful for detecting ALT, but without a metaphase spread it is difficult to judge total telomeric DNA content. A high-throughput technique favored by those carrying out large studies is quantitative PCR (qPCR) with two reactions - one with primers specific for telomeric sequence and one with a single copy gene to allow normalization [7,8].

The development of massively parallel sequencing, that is, next-generation sequencing, provides an alternative and potentially highly robust method to measure telomeres. Castle *et al*. [9] previously suggested a potential application for whole-genome sequencing (WGS) to ascertain telomeric DNA content. By counting and normalizing WGS reads containing the telomere repeats (TTAGGG)_4_, they reported that a lung carcinoid cell line had fewer telomere reads compared with the pooled DNA of healthy individuals [9]. This *in silico *finding, although consistent with the hypothesis that cell lines may have shorter telomeres due to many cycles of cell divisions, has several caveats. First, the observation was based on a single cell line with no experimental validation. Second, since the normal control DNA employed was not matched to the cell line source, it remains unclear if normal heterogeneity in telomere length might have contributed to the observed telomere difference. At present, the potential application of using WGS for telomere analysis has not been explored.

In this study we present the first comprehensive characterization of telomeres in primary tumors using WGS data from The St Jude Children's Research Hospital - Washington University Pediatric Cancer Genome Project (PCGP). The PCGP is sequencing 600 pediatric cancers and their matched normal DNA to identify somatic lesions that drive the initiation, biological and clinical behavior of pediatric cancers. It was launched in 2010 and WGS is complete for over 235 tumors from 15 different types of pediatric cancers with an average of 30-fold haploid coverage [10], making it possible to carry out a comprehensive telomere analysis using WGS data [11-14].

## Results and discussion

### WGS telomeric DNA content and age

**Figure 1:**
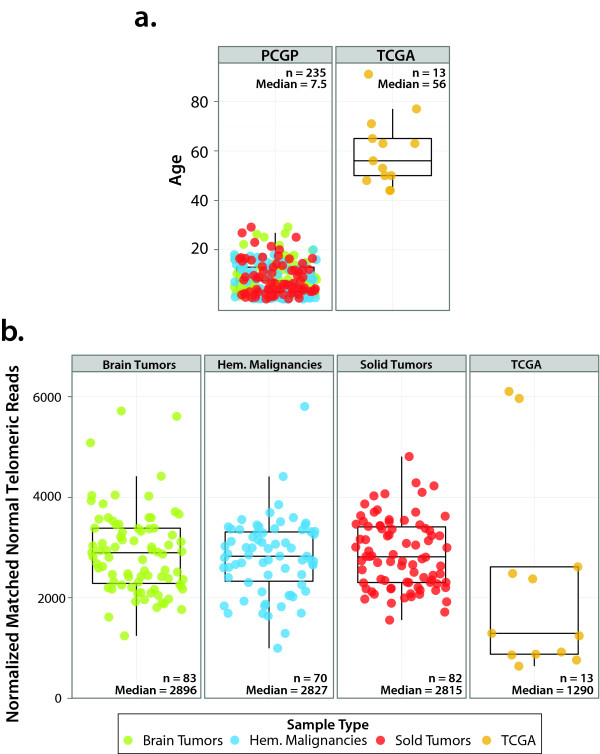
**Association with matched non-tumor telomeric DNA content and age**. **(a) **Comparison of age distribution of pediatric PCGP samples and adult TCGA samples. Because of the narrow range of age distribution in pediatric cancer in PCGP (n = 235, median = 7.5) we included 13 samples from TCGA (n = 13, median = 56) to enable evaluation of association between telomere length with age. **(b) **Comparison of distribution of normalized telomere count in matched normal DNA of pediatric patients with that of the adult patients. The reduction of telomere reads in adult is statistically significant (Wilcoxon signed rank *P *= 0.00046).

To evaluate the reliability of using WGS data for characterizing telomeric DNA content, we first compared the normalized count of reads containing telomere repeat (TTAGGG)_4 _of matched normal DNA from PCGP patients with that of normal DNA of 13 adult cancer patients from The Cancer Genome Atlas (TCGA). All samples used for this analysis were from either peripheral blood or bone marrow [15,16]. A reduction in telomere repeats with age is expected as telomeres erode at each normal somatic cell division. This result shows that the number of telomeric reads in adult sample was significantly lower than observed in three pediatric cancer groups (*P *= 0.02 by Mann-Whitney test; Figure 1), demonstrating that WGS is able to detect age-dependent changes in telomere length.

### WGS telomeric DNA content in matched tumor normal pairs

**Figure 2:**
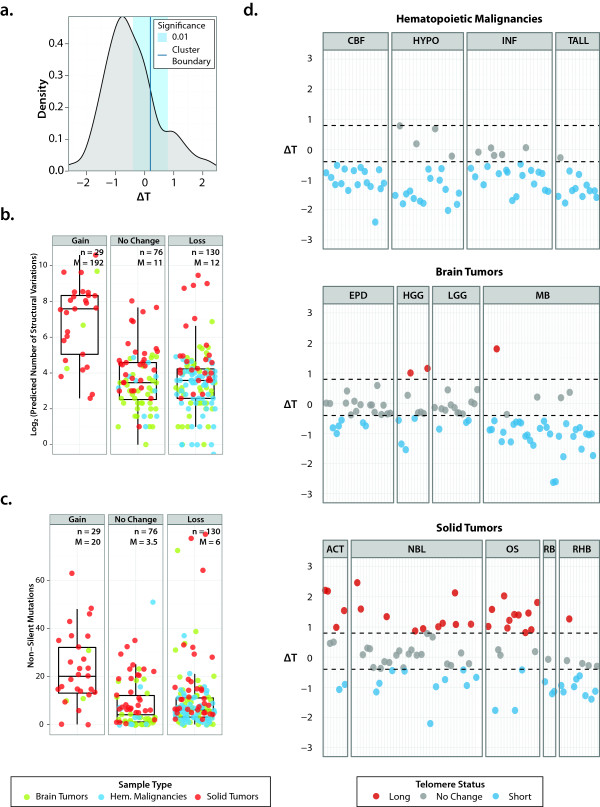
**Telomere analysis using whole-genome sequencing data of 235 pediatric cancers**. **(a) **Bayesian information criterion (BIC) guided clustering, which divided the ΔT values in this cohort into two clusters with equal variance. The boundary of these clusters is marked in dark blue. Using 0.01 as the threshold for significance, we defined the lower and upper boundary of ΔT as 'gain' or 'loss' of telomeric DNA. Samples that fall within these boundaries are deemed to have 'no change' in telomere status. **(b) **The number of structural variations in tumors with 'gain', 'loss' or 'no change' of telomere status. Tumors with ΔT gains have significantly higher number of structural variations compared with the other two groups (Mann-Whitney *P *= 1.07e-10; brain tumors *P *= 0.013, solid tumors *P *= 0.0002, hematopoietic malignancies *P *= NA (Not Applicable - no telomeric content gains detected); M, median). **(c) **The number of non-silent mutations in tumors with 'gain', 'loss' or 'no change' of telomere status. Tumors with ΔT gains have significantly higher number of sequence mutations compared with the other two groups (Mann-Whitney *P *= 3.723e-07; brain tumors *P *= 0.061; solid tumors *P *= 0.013, hematopoietic malignancies *P *= NA; M, median). **(d) **ΔT values from 235 pediatric cancers. The dotted lines correspond to the lower and upper boundary of ΔT as 'gain' or 'loss'. CBF, core-binding factor ALL; HYPO, hypodiploid ALL; INF, infant ALL; TALL, ETP-ALL; EPD, ependymoma; HGG, high-grade glioma; LGG, low-grade glioma; MB, medulloblastoma, ACT, adrenocortical carcinoma; NBL, neuroblastoma; OS, osteosarcoma; RB, retinoblastoma; RHB, rhabdomyosarcoma.

The original method by Castle *et al*. counted the number of reads containing (TTAGGG)_4 _and normalized to the average genomic coverage. We modified this approach by first normalizing the number of reads containing the telomeric sequence (TTAGGG)_4 _or its reverse complementary sequence (CCCTAA)_4 _relative to WGS average coverage, and then calculating the change in telomeric DNA content in tumor samples (ΔT) as the log2 ratio of the number of telomere reads in a tumor sample relative to a matched non-tumor sample from the same patient. This approach minimizes the effect of WGS coverage, a patient's age, and inter-individual telomere-length heterogeneity on the determination of telomeric DNA changes in tumor cells. To classify this change, we used Bayesian information criterion (BIC)-guided clustering of ΔT for all tumors to determine whether there was a gain, loss or no change in tumor telomere compared to normal DNA (Figure 2a).

We applied this method to 235 PCGP pediatric cancer genomes (Figure 2d) comprising 13 different cancer types. We found significant gains of telomeric DNA in 32% of solid tumors. In contrast, hematopoietic malignancies show near uniform loss of telomeric DNA and only 4% of brain tumors have telomere gain. Specifically, all of the core binding factor (CBF) acute myeloid leukemia (AML) tumors were found to have loss of telomeric DNA, 79% of hypo-diploid (HYPO) acute lymphoblastic leukemia (ALL), 77% of infant (INF) ALL, and 92% of early T-cell precursor ALL (ETP ALL ) had loss, while the remaining tumors of the hematopoietic malignancies group had no change in telomeric DNA. In brain tumor the majority (72%) of ependymoma (EPD) samples had no change in telomeric DNA while the remainder had loss. A similar pattern was observed in low-grade gliomas (LGG) as 80% of tumors had no change and 20% had loss. By contrast 85% of medulloblastoma had loss of telomeric DNA but one outlier (SJMB004, discussed below) had marked gains in telomeric DNA. High-grade gliomas had gains in 27%, losses in 36% and no change in 36% of tumors. The following members of the solid tumor malignancies had more gains in telomeric DNA: adrenocortical carcinoma (ACT, 50%), neuroblastoma (NBL, 27%) and osteosarcoma (OS, 61%). However, the dominant pattern in rhabdomyosarcoma (RHB) and retinoblastoma (RB) was loss of telomeric DNA, 62% and 75%, respectively. Telomeric DNA content for several pediatric tumors were previously studied and our results support previously published findings (comprehensively reviewed in [17]), that is, leukemia had shorter telomeres with no evidence for ALT [18,19], some of the ACT had very long telomeres indicative of ALT [19,20], a high proportion of osteosarcoma had long heterogeneous telomeres with ALT [21-23], NBLs had highly variable telomere lengths [24], and some of the high-grade gliomas had ALT and long telomeres [25]. This concordance provides a strong indication that telomeric DNA content measurement by WGS is applicable to multiple tumor types.

To evaluate the variability of telomere content estimations from WGS, we determined the telomeric DNA content for two infant ALL tumors that occurred in a pair of twins. Both tumors share the same initiating translocation of myeloid/lymphoid or mixed-lineage leukemia (MLL) gene, confirming that the twin pairs of leukemia have a common clonal origin, as expected from previous research on twins with concordant leukaemia [26]. Therefore, the twin pair of tumors can be considered a biological replica given their common clonal origin. The normalized read count for each tumor in the twin pair was very similar (2,266 versus 2,545), showing high reproducibility of telomere analysis by our approach (Figure S1 in Additional file 1).

**Figure 3:**
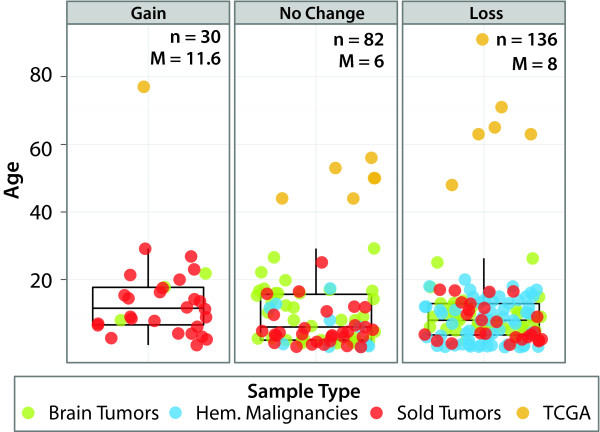
**Age distribution versus telomere status**. Age distribution in samples with different telomere status in tumor, that is, gain, loss and no change. There is no significant difference in age distribution across the three groups (ANNOVA, *P *= 0.368).

We examined telomere changes in the context of other somatically acquired genomic aberrations. Interestingly, tumors with telomere gains also contained a significantly higher frequency of genomic structural variations, which include deletions, inversions, insertions, intra- and inter-chromosomal rearrangements (Figure 2b; Mann-Whitney *P *= 1.07 × 10^-7^). Additionally, they had significantly higher numbers of non-silent somatic sequence mutations (Figure 2c; Mann-Whitney *P *= 3.723 × 10^-7^) compared with those with no change or loss of telomeres. When considering only brain tumors or solid tumors (no telomere gain in hematopoietic malignancies), telomere gain was significantly associated with structural variations (*P*-values for brain and solid tumor are 0.013 and 0.0002, respectively) but the association with sequence mutations was observed only in solid tumors (*P *= 0.013; brain tumors *P *= 0.061). In contrast to non-tumor DNA, no significant relationship was observed between patient age and telomere gain or loss status in tumors (Figure 3).

### Validation of telomeric DNA content predictions

**Figure 4:**
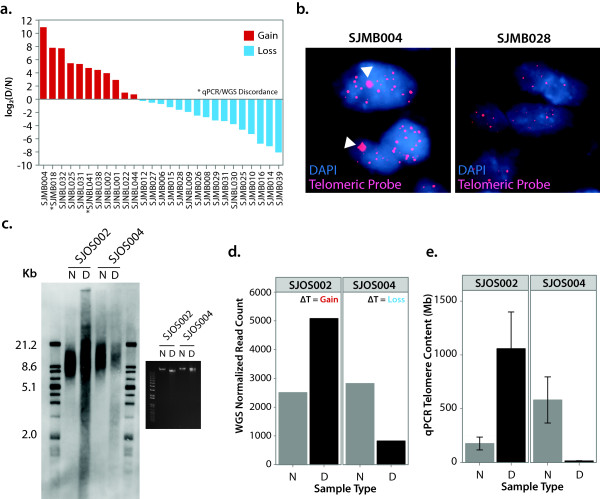
**Validation of WGS telomeric DNA content predictions**. **(a) **Quantitative PCR for a subset of samples, including 16 medulloblastoma and 11 neuroblastoma samples, showing changes in telomeric DNA content between normal and diagnosis log_2_(Absolute telomere length D/Absolute telomere length N. **(b) **FISH using probes for telomeric DNA confirms the 'normal' and 'abnormal' (ultra-bright spots, white arrowheads) telomere patterns in SJMB028 and SJMB004, respectively. **(c) **Telomere restriction fragment (TRF) analysis by southern blotting in 2 osteosarcoma samples, SJOS002 and SJOS004 predicted to have telomere gain and loss by WGS, respectively. (N = matched normal DNA, D = diagnosis tumor DNA). Inlay is the 100ng of genomic DNA on a 0.5% ethidium bromide gel which shows that the DNA quality is acceptable. **(d) **WGS normalized telomeric read counts for SJOS002 and SJOS004. **(e) **qPCR measurement of absolute telomeric DNA in SJOS002 and SJOS004 (error bars represent standard deviation of three technical repeats).

To validate our method of measuring telomere length from WGS data, we used three independent assays of telomere length. First, quantitative PCR [27] was used to validate telomere change identified by analysis of WGS data in 25 tumors using DNA from both tumor and matched non-tumor samples. We used ΔT_qPCR _, that is, the log2 ratio of total absolute telomere quantity in tumor compared to non-tumor based on qPCR and found 88% of the tumors show consistent telomere status between WGS and qPCR (Figure 4a; Table S1 in Additional file 2). Second, 30 samples were subjected to interphase FISH analysis. Using this method, it was possible to determine only 'normal' or 'abnormal' telomeres. Therefore, telomere gain by WGS analysis is considered 'abnormal' while the remaining cases are considered 'normal'; 87% of the telomere status predictions by WGS were concordant with telomere FISH (Table S1 in Additional file 2). SJMB004, a medulloblastoma with a marked gain of telomeric DNA by WGS, had large ultra-bright telomere foci expected from cells utilizing ALT (Figure 4b). In contrast, ALT was absent in SJMB028, a sample predicted to be 'normal' by WGS. The third validation experiment was TRF analysis by southern blotting on two samples with sufficient quantity of genomic DNA, SJOS002 and SJOS004 (Figure 4c). SJOS002 and SJOS004 were predicted to have gain and loss of telomeric DNA by WGS (Figure 4d) and qPCR (Figure 4e), respectively, and TRF analysis supports these findings.

## Conclusions

Our study is the first application of WGS to measure telomeric DNA content in a large collection of primary tumors. Our extensive validation shows that WGS analysis is a reliable approach for determining telomeric DNA content changes in cancer genomes. It should be noted, however, that telomeric DNA content assessment by WGS has comparable pitfalls to those of qPCR, that is, chromosome by chromosome telomere length cannot be quantified and contribution of telomeric repeats in non-telomeric regions of the genome cannot be determined. Our findings not only corroborate previous reports of telomeric DNA content in several pediatric cancers [17], they also add a significant amount of telomere status information to tumors that have not been adequately studied. Furthermore, integrating tumor telomeric state ('gain', 'loss', or 'no change') with other somatic lesions such as sequence mutations and structural variations in one single WGS experiment provides additional insight into the landscape of genetic alterations in cancer. For example, significant association between telomere change and structural variation suggests that telomere gain could be a hallmark of genome instability. Integrating sequence mutations with the telomere features may identify causal mutations for the abnormal telomere phenotypes. For example, we have previously reported that mutations in *ATRX *are linked to telomeric gains in NBL [12]: of the ten NBL tumors with *ATRX *somatic alterations, eight have longer telomeres. Although our analysis was based on WGS, we anticipate that similar approaches can be applied for transcriptome sequencing (RNA-seq) data by analyzing aberrantly expressed telomeric DNA.

## Materials and methods

### Patients and samples

The use of human tissues for WGS was approved by the institutional review boards of St Jude Children's Research Hospital, Memorial Sloan-Kettering Cancer Center, and Washington University in St Louis (St Jude IRB# FWA00004775, Protocol# XPD09-018). Written informed consent and/or assent was obtained from patients and/or legal guardians at the time of the surgical resection or bone marrow procedure. Matched normal samples were obtained either from peripheral blood, bone marrow or adjacent normal tissue.

### Whole genome sequencing

Illumina 100 bp paired-end sequencing was performed for tumor and normal DNA from 235 subjectsat a high average genomic coverage (approximately 30×). Single nucleotide variations, insertion/deletions, were detected by the program Bambino [28] followed by an automated review process as described previously [11]. Structural variations were identified by CREST [29]. The WGS data used in this study have been deposited at the European Genome-phenome Archive (EGA) and data from all of the diseases examined in this manuscript can be found at [30]. Information on data access policies can be found at [31]. Table 1 lists dataset IDs.

**Table 1:** Whole genome sequencing data sets used for telomere analysis

Study	Dataset ID
Retinoblastoma	EGAD00001000261
ETP-ALL	EGAS00001000348
Neuroblastoma	EGAD00001000135
Medulloblastoma	EGAD00001000269
Osteosarcoma	EGAD00001000159
Aderenocortical carcinoma	EGAD00001000160
Rhabdomyosarcoma	EGAS00001000256
Low-grade glioma	EGAD00001000161
Edendymoma	EGAD00001000162
Infant-ALL	EGAD00001000165
High-grade glioma	EGAD00001000085
Hypo-diploid ALL	EGAD00001000260
Core binding factor ALL	EGAD00001000268

The ETP-TALL data set has just become public in EBI under the accession EGAS00001000348. We updated the accession in Table 1.

### Assessment of telomeric DNA content using whole genome sequencing

Reads containing the telomeric repeat (TTAGGG)_4 _or (CCCTAA)_4 _were counted and normalized to the average genomic coverage (that is, the average number of average reads covering each base in the reference human genome). The normalized telomere count was obtained separately for each tumor and its matching normal WGS. From this the log_2 _ratio was calculated giving ΔT. Adjustment for GC bias is not required because at an average of 43% GC content for telomeric reads no bias is expected for calibrating DNA abundance by Illumina sequencing reads [32].

### Classification of telomere change in tumors

Based on the data produced, we observed that a number of samples had a very small ΔT (close to 0) and postulated that this may reflect random variation in the telomere counts produced by library preparation or sequencing bias. Therefore, we performed Gaussian mixture modeling on the ΔT values using the mclust package [33] (version 3.4.8) in R-2.11.1. The optimal model according to BIC contains two clusters, those samples with gain and loss of telomeric DNA. Based on this modeling we were able to classify the samples into three groups: 1) samples that reject the null hypothesis that the data come from the second cluster at a significance level of 0.01. ('gain' of telomeric DNA); 2) samples that reject the null hypothesis that the data come from the first cluster at a significance level of 0.01. ('loss' of telomeric DNA); 3) remaining samples ('no change' in telomeric DNA).

### FISH for telomeric DNA

Interphase FISH was performed on 4-µm-thick, formalin-fixed, paraffin-embedded tissue sections. The Cy3-labeled TelG probe (PNAbio, Thousand Oaks, CA, USA) was co-denatured with the target cells on a hotplate at 90°C for 12 minutes. The slides were incubated for 48 hours at 37°C and then washed in 4 M Urea/2× SSC at 45°C for 5 minutes. Nuclei were counterstained with DAPI (200 ng/ml; Vector Labs, Burlingame, CA, USA).

### Quantitative PCR measurement of absolute telomere length

qPCR was carried out as described previously [8,12]. Diagnostic and matched normal whole genome amplified DNA (15 to 20 ng) was each subject to qPCR in two reactions on the sample 96-well plate, one to amplify telomeric sequence and one to amplify a common gene, *RPLP0*. All reactions were carried out using Brilliant III Ultra-Fast SYBR Green master mix (Agilent) on a Stratagene Mx3000 thermal cycler with the following conditions; 95°C for 10 minutes followed by 40 cycles of 95°C for 15 s and 60°C for 1 minute.

### Telomere restriction fragment analysis

Southern blotting was performed using the *T*elo*TAGGG *kit (Roche Diagnostics, Indianapolis, IN, USA - 12209136001 v8.0, ). Restriction digested genomic DNA (1.5 μg) was loaded onto a 15 cm 0.8% ultra pure agarose gel and run for 2 to 4 hours at 75v. The gel was incubated in hydrocholoric acid solution, denatured and neutralized before transferring overnight to a positively charged nylon membrane with 20× SSC using a Whatman (Maidstone, Kent, UK) TurboBlotter. The DNA was fixed by exposing the membrane to UV. The membrane was pre-hybridized for 60 minutes at 42°C in DIG Easy Hyb solution before hybridization for 3 hours at 42°C with a telomere probe (10 μl probe in 10 ml pre-warmed DIG Easy Hyb). The membrane was washed and then blocked and incubated with anti-DIG antibody; after another round of washing the signal was detected using the supplied substrate solution and exposed to X-ray film.

### Calculation of validation rates

For qPCR, validation rates were calculated for those samples that had either 'loss' or 'gain' of telomeric DNA. Those with 'no change' were excluded due to the ambiguity of their result. For FISH analysis validation rates were calculated as follows. FISH assessment of telomere normality gives two classes of sample, 'normal' and 'abnormal'; no change or loss of telomeric DNA as called by WGS would appear 'normal' by FISH and those samples with gain of telomeric DNA by WGS would be classified as 'abnormal' by FISH.

### Statistical analysis

Statistical analysis was performed using R (version 2.11.1) and plots generated using the ggplot2 package.

## Abbreviations

ACT: adrenocortical carcinoma; ALL: acute lymphoblastic leukemia; ALT: alternative lengthening of telomeres; BIC: Bayesian information criterion; FISH: fluorescence *in situ *hybridization; NBL: neuroblastoma; PCGP: Pediatric Cancer Genome Project; qPCR: quantitative PCR; TCGA: The Cancer Genome Atlas; TRF: telomere restriction fragment; WGS: whole-genome sequencing.

## Competing interests

The authors declare that they have no competing interests.

## Authors' contributions

MP contributed to the conception of the study and carried out the experiments, including bioinformatic analyses and laboratory validation of the findings. XC, MR and GW contributed to bioinformatics and statistics, including clustering of ΔT, read mapping and coverage analysis, respectively. AB, JD and DE carried out FISH experiments. Experimental design and execution were overseen by JE. JZ designed the study and wrote the manuscript. NKC, MD, EM, RW, CM, RG, SB, GZ and JRD were involved in drafting and critical review of the manuscript. All authors read and approved the final manuscript.

## Supplementary Material

Additional file 1 **Supplementary figures**. Click here for file

Additional file 2 **Telomere calls for WGS, qPCR and FISH (where appropriate) for all of the samples analyzed in this manuscript**. Click here for file
