# Supplementary material for: Assessing telomeric DNA content in pediatric cancers using whole-genome sequencing data
Source: Genome Biol. 2012 Dec 11;13(12):R113. doi: 10.1186/gb-2012-13-12-r113 (PMC3580411; doi:10.1186/gb-2012-13-12-r113)
Supplement: Additional file 1 — Supplementary figures. [file gb-2012-13-12-r113-S1.DOCX]

Supplementary Information for…

# Assessing telomeric DNA content in pediatric cancers using whole-genome sequencing data.


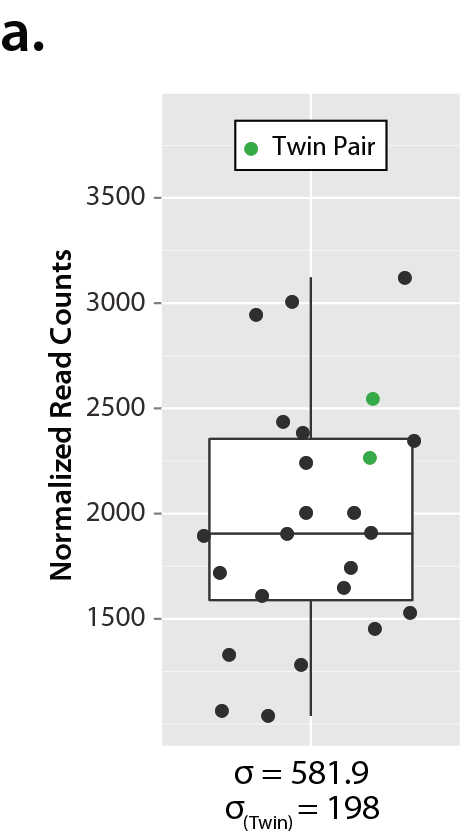


**Figure S1** – Biological Reproducibility

**A.** Infant ALL normalized diagnostic telomere read counts including a set of twins. Both of these twins contain the same initiating MLL translocation event (data not shown) indicating that their tumors share a common clonal origin. The standard deviation telomere content in these two samples is under half of the standard deviation for the whole cohort.
